# Non-alcoholic Fatty Liver Disease and Liver Fibrosis during Aging

**DOI:** 10.14336/AD.2022.0318

**Published:** 2022-07-11

**Authors:** Yuan Li, Nia T. Adeniji, Weiguo Fan, Koshi Kunimoto, Natalie J. Török

**Affiliations:** Gastroenterology and Hepatology, Stanford University, Palo Alto, CA 94305, USA

**Keywords:** liver, NAFLD, fibrosis, aging, senescence

## Abstract

Non-alcoholic fatty liver disease (NAFLD) and its progressive form non-alcoholic steatohepatitis (NASH) have emerged as the leading causes of chronic liver disease-related mortality. The prevalence of NAFLD/NASH is expected to increase given the epidemics of obesity and type 2 diabetes mellitus. Older patients are disproportionally affected by NASH and related complications such as progressive fibrosis, cirrhosis and hepatocellular carcinoma; however, they are often ineligible for liver transplantation due to their frailty and comorbidities, and effective medical treatments are still lacking. In this review we focused on pathways that are key to the aging process in the liver and perpetuate NAFLD/NASH, leading to fibrosis. In addition, we highlighted recent findings and cross-talks of normal and/or senescent liver cells, dysregulated nutrient sensing, proteostasis and mitochondrial dysfunction in the framework of changing metabolic milieu. Better understanding these pathways during preclinical and clinical studies will be essential to design novel and specific therapeutic strategies to treat NASH in the elderly.

An aging world population with an increased prevalence of comorbidities, such as obesity, type 2 diabetes (T2DM), and hypertension, has led to the emergence of non-alcoholic fatty liver disease (NAFLD) as the leading cause of chronic liver disease-related mortality [1,2]. In the United States, relative to the period 1988-1994, there has been more than a 160% increase in NAFLD prevalence [3]. The current global prevalence is 24% and is estimated to impact 100 million patients in the United States by the year 2030 [1,4]. About 20-30% of patients with NAFLD can progress to non-alcoholic steatohepatitis (NASH) characterized by steatosis, necroinflammation, hepatocyte ballooning, and in advanced cases, fibrosis. Clinically, patients with NAFLD/NASH are often asymptomatic and are diagnosed late in the disease process, when advanced fibrosis is established or when complications due to portal hypertension or hepatocellular carcinoma (HCC) arise [5]. Unfortunately, there is no approved treatment, and liver transplantation remains the only curative option for most patients. However, older patients often do not qualify because of frailty or comorbidities. Advanced fibrosis is more common in the elderly [6,7], and the presence of stage 3-4 fibrosis is the strongest predictor of liver-related and all-cause mortality in NASH [8]. In this review, our goal is to focus on specific dysregulated processes in the aging liver that could drive fibrosis and outline potential areas for future studies and treatment approaches.

## Senescence and Aging in NAFLD/NASH

Replicative senescence is triggered by age-induced telomere shortening/erosion [9] or deficiency [10], while stress-induced premature senescence occurs under external or intracellular sublethal stress causing oxidative DNA damage [11]. Senescent cells are generally characterized by cell cycle arrest, DNA damage response [e.g., presence of gamma H2A histone family member X (γH2AX) foci], and elevated senescence-associated β-galactosidase activity [10,12]. The cells may have facultative heterochromatin enriched with histone modifications, known as senescence-associated heterochromatin foci [13]. They may acquire a senescence-associated secretory phenotype (SASP) with abundant secretion of interleukins (e.g., IL-1α, IL-6), inflammatory cytokines & chemokines [e.g., C-C motif chemokine ligand (CCL2)], growth factors, and proteases [e.g., Matrix metallopeptidase 1 and 3 (MMP-1, MMP-3)] [14]. However, the specific SASP changes are often cell-type- and/or microenvironment-dependent [15].

The role of senescence and aging in NAFLD/NASH are relatively new areas of investigation. Interestingly, telomere length seems to be preserved in hepatocytes and cholangiocytes in healthy livers during aging [16]. However, in NASH patients, biopsies exhibit shorter telomeres, a high level of p21 indicating cell cycle arrest at the G1/S phase, and an increased DNA damage response with the presence of γH2AX. Higher hepatocyte p21 expression in hepatocytes correlated with disease stage, T2DM, and adverse outcomes [17,18]. In a different study p21 gene polymorphism appeared to affect the development but not the progression of fibrosis [19]. In a 6-year cohort study, in patients with T2DM who developed NASH, telomere shortening was observed in peripheral blood leukocytes, which was associated with an increased risk for progressive fibrosis [20]. These data included subjects of various ages. Therefore, it would be important to conduct longitudinal studies to evaluate if hepatocyte telomere shortening is an independent risk factor for more aggressive NASH in the elderly.

In an animal model of telomerase reverse transcriptase (Tert)-deficiency there was a failure to activate genes involved in the metabolic response to a high-fat diet leading to hepatocyte injury and steatosis. Interestingly, glucose metabolism was not altered in these mice; however, in *Tert^-/-^* hepatocytes the citric acid cycle (TCA) was dysregulated, with altered reduced nicotinamide adenine dinucleotide phosphate (NADPH)/ nicotinamide adenine dinucleotide phosphate (NADP^+^) ratios. Chemical inhibition of telomerase recapitulated the phenotype. These studies highlight the metabolic role of telomere enzyme dysfunction [21]. Destroying senescent cells by suicide gene-meditated ablation of p16^Ink4a^-expressing cells or using senolytic drugs improved steatosis in two NASH models [18,22]. Targeting the urokinase-type plasminogen activator receptor (uPAR, that was induced during senescence) by Chimeric antigen receptor (CAR)-T cells, efficiently ablated senescent cells *in vitro* and *in vivo*, improving liver fibrosis in a NASH model [23]. Also, a recent study demonstrated that senescent cells/SASP-driven proinflammatory M1 macrophages exhibit an increased cluster of differentiation (CD38) expression, and enhanced nicotinamide adenine dinucleotide nucleosidase (NADase) activity, resulting in low NAD^+^ in the liver [24]. To increase NAD^+^ levels *in vivo*, natural NAD^+^ precursors, such as nicotinamide riboside could be used and converted into bioavailable NAD^+^. Nicotinamide riboside treatment could improve mitochondria-specific unfolded protein response (UPR) and ameliorate liver steatosis and injury in NASH [25].

Senescent hepatic stellate cells (HSC), the primary liver's primary fibrogenic cells, have been investigated in several recent studies. Senescence-activated HSC accumulate in fibrotic livers and represent a proinflammatory and anti-fibrogenic phenotype, with decreased secretion of extracellular matrix (ECM) components, and an increase in ECM-degrading enzymes [26]. Activity of the key senescence regulator p53 was essential for senescence, and HSC lacking p53 continued to proliferate propagating fibrosis in the CCl_4_ model [27]. Furthermore, Insulin-like growth factor 1 (IGF-1) treatment induced HSC senescence in a different model, limiting fibrosis in a p53-dependent manner [28]. These studies point to the essential physiological role of HSC senescence limiting fibrosis progression albeit with a proinflammatory effect. These findings should be further substantiated in NASH models in aged animals.

Another critical aspect of HSC senescence is that it could be linked to carcinogenesis in NASH [29]. In the study by Yoshimoto *et al.* an increase in the gut microbiota-derived deoxycholic acid promoted SASP by HSC. Blocking deoxycholic acid production or depleting senescent HSC prevented HCC progression [29]. In a recent study evaluating the BET family protein degrader as a potential senolytic drug showed promise in eliminating senescent HSC in steatotic livers, and reduced tumorigenesis [30]. In human NASH-related HCC, cancer-associated fibroblasts (CAF) demonstrated increased expression of SASP compared to those from HCC of other etiologies such as viral hepatitis [31]. These findings highlight a critical role of senescent HSC/CAF in promoting tumor growth, but further work is needed to consider the heterogeneity of HSC and CAF. Developing senolytics is an intense area in drug development [32]. However, in NASH/aging, more mechanistic studies are needed to elucidate the bidirectional crosstalk of senescent hepatocytes and HSCs and the effects on the immune microenvironment to avoid potential off-target effects.

## Nutrient Sensing and Proteostasis in Aging and NAFLD/NASH

### Nutrient sensing

Cells sense nutrients either by engagement of specific sensors or by indirect detection of surrogate metabolites. Several nutrient-sensing pathways are key to aging-related diseases, including the mammalian target of rapamycin (mTOR) [33-35], adenosine monophosphate-activated protein kinase (AMPK) [36], general control nonderepressible 2 (GCN2) [33], and IGF-1 [34,37]. Rapamycin has been reported to improve the health-span of aging mice [38], and using rapamycin accelerated hepatic protein turnover and attenuated oxidative stress [39,40]. Even though rapamycin is an FDA-approved drug with considerable clinical experience and despite several positive studies in murine models linking it to an increase in lifespan [41,42], no studies have yet been able to confirm a clear effect in patients.

AMPK plays a central role in regulating energy homeostasis [43]. Although studies in the aging population with NASH are still limited, it is tempting to speculate that dysregulated AMPK activation and responsiveness are key to aberrant nutrient sensing [44]. Liver-specific AMPK knockout (AMPK KO) exacerbated diet-induced NASH in mice, and genetically or pharmacologically activating AMPK corrected NASH-related inflammation, steatosis, and fibrosis [45-47]. AMPK KO increased caspase 6 activation, generating a feedforward loop to sustain the caspase cascade and apoptosis. Activation of AMPK or inhibition of caspase-6 improved liver damage and fibrosis in NASH [46]. These studies suggest that AMPK is a druggable target for NASH, though achieving liver specificity is still a significant challenge. AMPK/mTOR could also be targeted by peroxisome proliferator-activated receptor (PPAR) δ, reducing the intrahepatic lipid content and stimulating β-oxidation involving an autophagy-lysosomal pathway [48]. As PPARδ activators (PPARα/δ, Elafibranor) are being investigated in NASH in a current Phase 3 trial, extending these studies to the aging NASH population would be interesting.

GCN2 is central to sense stress signals such as amino acid starvation and coordinates nutrient sensing and redox responses. In aged mice, GCN2-deficiency exacerbated fat consumption at the expense of carbohydrate intake and prevented increased protein consumption [49]. Therefore, GCN2 signaling might be an ancient pathway contributing to macronutrient selection and food preference. GCN2-deficient mice are partially protected from high-fat-diet-induced liver dysfunction, steatosis, and insulin resistance [50], although these studies were done in young mice. GCN2 can also control oxidative stress via the regulation of glutathione peroxidase 1 and the amount of carbonyl radicals in the liver [51]. In the gut, GCN2 protects against inflammasome activation [52]. Given the role of GCN2 in several organs, its role in NAFLD/NASH in aged subjects would require further investigation.

In addition to the pathways mentioned above, Sirtuins (SIRTs), NAD^+^-dependent deacetylases, have emerged as important sensors/regulators of metabolic pathways, especially during aging. Studying the age-dependent metabolic changes in the circadian hepatic transcriptome in young and old mice, calorie restriction was shown to improve NAD^+^ availability, SIRT1 activity, and restore global protein acetylation over the circadian cycle in old mice [53]. In the context of NASH, systematic SIRT1 ablation or hepatocyte SIRT1 deletion led to hepatic steatosis and inflammation [54,55], and SIRT1 overexpression protected against diet-induced steatosis [56,57]. Taken together, restoring dysregulated SIRT1 activity potentially could reduce NASH progression in older patients.

### Proteostasis

The proteostatic process is regulated by multiple mechanisms including chaperons, the ubiquitin-proteasome, and the autophagy-lysosome systems [58]. *Autophagy* is generally considered an evolutionarily conserved adaptive process when cells eliminate potentially toxic material in response to stress. It can be classified into macroautophagy, microautophagy, and chaperone-mediated autophagy (CMA). CMA controls the degradation of selective proteins into lysosomes and is impaired during aging, resulting in reduced proteostasis and stress resistance [59]. Modulating the lysosomal receptor for CMA that was shown to decrease with age reversed CMA and improved liver function [60]. It is also notable that while the dysregulated CMA function could be compensated by other proteolytic systems in young animals, a decline in this compensation during aging led to perturbed proteostasis and stress resistance [61].

Other forms of autophagy, macroautophagy and its specialized form lipophagy involved in the degradation of intracellular lipids, are also downregulated in hepatocytes in NASH, and excellent reviews are summarizing the key pathways [62,63]. It is possible that in older animals with NASH these pathways could be further exacerbated, and interventions such as calorie restriction, physical exercise, and genetic/pharmacologic induction of autophagy could restore liver proteostasis and could be useful in treating NASH in aged subjects. Indeed, intermittent calorie restriction was shown to improve NAFLD in aged mice [64]. Caffeine that modulates lipophagy and mitochondrial β-oxidation was shown to have hepato-protective effects in NAFLD [65]. Furthermore, several pharmacological activators of autophagy exerted beneficial effects, including ezetimibe that can induce AMPK activity [66], glucagon-like peptide 1 (GLP-1) analogues that can promote macroautophagy [67], and trehalose that can inhibit glucose transport, and also induce Sequestosome 1 (p62) upregulation activating the Kelch-like ECH-associated protein 1 (Keap1)-Nuclear factor-erythroid factor 2-related factor 2 (Nrf2) pathway with anti-oxidant effects [68].

When the protein folding capacity of ER is exceeded, known as “ *endoplasmic reticulum (ER) stress*”, accumulation of unfolded or misfolded proteins could elicit signal transduction pathways known as UPR to restore the protein homeostasis [69]. UPR is activated by three sensors including inositol-requiring protein 1, protein kinase RNA-like ER kinase, and activating transcription factor 6, and maladaptive UPR leads to apoptosis [70]. ER stress-related proteins increase in the liver during aging, associated with increased hepatic insulin resistance, and increased liver glucose in response to pyruvate challenge and hyperglycemia in old rats [71]. Obesity-induced ER stress was shown to impair insulin resistance via downregulating liver X-box binding protein 1, hyperactivating c-Jun N-terminal kinase, and subsequently phosphorylating the insulin receptor substrate 1 [72]. ER stress was linked to the downregulation of the farnesoid X receptor, a key regulator of hepatic lipid metabolism via the inhibition of hepatocyte nuclear factor 1α transcriptional activity, exacerbating age-related steatosis [73]. ER stress was also shown to promote fibrosis via inducing hepatocyte apoptosis leading to myofibroblast activation, and macrophage polarization [74], though further direct evidence in NAFLD/NASH and aging is still required.

## Mitochondrial Dysfunction in Aging and NASH

Age-related mitochondrial changes may contribute to the progression of NAFLD/NASH in patients, though direct evidence is still limited. Assessment of the mitochondrial functional status and adaptive events are complex, and results can vary upon the methods used, age differences, gender, and stages of disease. Therefore, it is not surprising that there are contradicting observations about the role of mitochondrial function in aging and NAFLD/NASH [76,77].

### Mitochondrial quality

Mitochondrial quality control involves several processes such as proteostasis, discussed earlier, biogenesis, dynamics, and mitophagy, reviewed in Zhou *et al.* recent publication [78]. During aging there is a slow turnover of mitochondria due to dysregulated biogenesis, fission/fusion, and/or defective autophagic clearance. Mitofusin 2 (Mfn2) deficiency, for instance reduces phosphatidylserine transfer and phospholipid synthesis, thereby leading to ER stress and the development of a NASH and HCC [79]. Age-related Mfn2 decrease has been appreciated in the muscle [80], obesity, and T2DM [81]. Whether Mfn2 depletion really occurs during aging in the liver and is a causative factor in NASH, should be further investigated. Mitophagy is a mitochondria-specific form of autophagy, and is considered as a protective mechanism in NAFLD/NASH [82]. Due to the dynamic nature of autophagy/mitophagy, accumulation of substrates like p62 and microtubule-associated proteins 1A/1B light chain 3B (LC3) could result from induced autophagy initiation or inhibited autophagy degradation, so one must be cautious with interpreting the autophagy/mitophagy flux status especially *in vivo* [83]. Nevertheless, several pathways could be involved linking defective mitophagy to NASH and aging. LC3 receptors that are located on the mitochondria can directly bind to LC3 and recruit damaged mitochondria to the autophagosomes. For instance, proteins such as Nip3-like protein X (NIX) and BCL2/Adenovirus E1B 19 kDa Interacting Protein 3 (Bnip3) can interact with LC3 and play a role in regulating mitochondrial integrity and lipid metabolism [84].

### Mitochondrial metabolism

There is also ample evidence that during aging mitochondrial metabolism is altered, and related transcripts [e.g. oxidative phosphorylation (OXPHOS), fatty acid (FA) β-oxidation, mitochondrial biogenesis] [85], and respiratory control ratio, indicating mitochondrial function, decrease with age [86]. High-fat diet decreases OXPHOS activity and destabilizes its subunits, contributing to impaired electron transport chain activities and ATP synthesis [87,88]. Mitochondrial β-oxidation is key to free fatty acid (FFA) metabolism into acetyl-CoA. Carnitine palmitoyl transferase 1 α (Cpt1a) and acyl-CoA oxidase 1 (Acox1) were downregulated during aging [89], owing to the decreased transcriptional activity of PPARα. Mice with hepatocyte-specific PPARα KO had impaired FA catabolism, leading to lipid accumulation even on a standard chow diet during aging [90]. Whether HSC are activated in this model would need to be further investigated, especially that PPARα agonist was shown to reduce inflammation and fibrosis in NASH in younger animals [91]. PPARα downregulation could also be linked to the advanced glycation end product receptor (RAGE) in older mice, and its downregulation improved PPARα, mitochondrial β-oxidation and steatosis [78].

Perturbed mitochondrial β-oxidation leads to lipid peroxidation with diverse toxic effects in NASH [92]. A study in patients revealed that mitochondrial function initially shifts to adapting to the increasing bioenergetics demand (“hepatic mitochondrial flexibility”), but then continued FFA overload overwhelms both TCA and FA catabolism, and this adaptation is lost in late NASH [93]. Whilst this study was performed in younger patients, it is likely that the maladaptive responses to FFA load are more enhanced during aging, and this would require carefully designed studies in older patients with NASH. Oxysterols, the oxidative products of cholesterol are also linked to decreased respiration in isolated liver mitochondria, down-regulation of transcription factors involved in mitochondrial biogenesis and to apoptosis of hepatocytes. This in turn can trigger the HSC activation and liver fibrosis [94,95].

### Mitochondria-associated membranes (MAM)

MAM, also known as mitochondria-endoplasmic reticulum contact sites, refer to an ER region dynamically tethered to the mitochondria. This subcellular compartment is important in the communication between mitochondria and ER, regulating Ca^2+^ trafficking, lipid metabolism, redox signaling, autophagy/mitophagy [96]. Dysfunctional MAM is implicated in senescence, and aging [97,98], and could thus be involved in NASH pathogenesis *via* the following mechanisms: (1) enriched MAMs can lead to Ca^2+^ overload thus compromising mitochondrial function [99,100]; (2) impaired MAM integrity could interrupt hepatic glucose sensing and insulin sensitivity [101,102]; (3) impaired MAM integrity may disable mitochondrial adaptation in response to glucose availability [102]. Drugs like rosiglitazone, troglitazone, and metformin show therapeutic effects on NAFLD in rodents restoring their MAM composition or function [103].

### Mitochondrial DNA (mtDNA)

Finally, aging is known to correlate with accumulating mtDNA mutations, deletions [104,105], and decreased mtDNA copy numbers [106]. Mice that express a proofreading-deficient form of a nuclear-encoded mitochondrial DNA polymerase, display a higher number of mitochondrial point mutations and deletions, as well as shortened life span [107]. Increased circulating mtDNA has been observed in older patients [108], and in NAFLD/NASH patients it was positively associated with disease severity [109]. Hepatocyte-derived mtDNA during NASH is linked to sterile inflammation in a Toll-like receptor 9 (TLR9)-dependent manner [110], and was also shown to drive liver fibrosis by activating HSCs [109].

## Liver Fibrosis and Aging in NASH

Aging is clinically associated with accelerated fibrosis in NASH patients [10,111-116]. Fibrosis strongly depends on the dynamic cell-cell communication in the fibrotic niche [117], involving crosstalk between hepatocytes, HSC, liver sinusoidal endothelial cells (LSECs), cholangiocytes, and immune cells. There are several excellent reviews discussing liver fibrosis in the context of aging [118,119]. Here our goal is to highlight pathways that could be pertinent to NASH, focusing on HSC and LSECs.

*HSC* are activated in response to injury and differentiate into alpha-smooth muscle actin (αSMA)-expressing, proliferating, and migrating myofibroblast-like cells that synthesize ECM proteins. In several mouse models activated HSC undergo senescence, characterized by decreased proliferation, ECM production and induced SASP components, including fibrolytic MMPs and cytokines, favoring immune clearance and thus fibrosis resolution [120]. Senescent HSC as mentioned earlier may play various roles in NASH: they could be protective against fibrosis while exacerbating inflammation [27,28] and could be linked to an increased risk of tumorigenesis [29]. Recently it was shown that in the aging liver, mechanosensing via integrin α_5_/β_1_ by HSC was dysregulated and HSC acquiring a senescent phenotype had reduced hepatocyte growth factor release impacting regenerative responses [121]. This highlights the multifaceted role of HSC that should be further explored in the context of aging.

During aging liver fibrosis/HSC activation-related transcripts were amongst the top canonical pathways that were induced in mice on high-fat diet [119]. Production of ROS is strongly linked to HSC activation [122], and NADPH oxidases (NOXs) are essential sources of superoxide or in the case of NOX4 hydrogen peroxide [123]. NOX1, NOX2, and NOX4 were identified as the most important homologues in the liver [124,125]. The nonphagocytic NOX1 and NOX4 are increasingly recognized as key enzymes in oxidative injury and wound healing, and they were found to be induced during aging [119,126], playing an important role in redox-mediated HSC activation [127,128]. On the other hand, the NOX2 complex can be induced in the aging hepatocytes in NASH in a non-phagocytic manner by directly binding the aging protein p52Shc to the p47^phox^subunit [129], thereby assembling and activating the enzyme complex leading to fibrosis. Targeting the NOX4-Nrf2 imbalance successfully reduced fibrosis in idiopathic pulmonary fibrosis [130] and NOX1/4 inhibition improved liver redox injury and fibrosis in NASH models [126,131], thus pharmaceutical approaches for NOX enzymes could be explored to treat aging-related fibrosis progression [132].

Long non-coding RNAs have been mapped in the aging liver and were found to be differentially regulated (e.g. *Meg3, Rian*, and *Mirg*) [133]. In other systems these were related to stress response, inflammatory and fibrogenic pathways. It would be interesting to see whether these can specifically be involved in NASH during aging, dysregulating fibrogenic activity.

There are fewer studies that focus on *fibrosis resolution* during aging. While developing drugs that limit HSC activation is an important goal, many patients with advanced fibrosis and crosslinked ECM may not benefit from these. Hence ECM turnover needs to be extensively studied during aging. There is experimental evidence that ECM remodeling is affected in old rats, type I and II collagen turnover was significantly reduced, whereas type IV and V collagen degradation biomarkers were induced [134]. In a CCl_4_-induced fibrosis model, in old mice fibrolysis was hampered due to reduced MMP13 and collagenase activity. In the same model, striking differences were observed in macrophage polarization, in young livers macrophages exhibited a remodeling phenotype, whereas macrophages from older livers had a pro-fibrogenic phenotype with higher Transforming growth factor beta (TGF-β) expression. [135]. Activation of Lysyl oxidase-like 2 (LOXL2), Transglutaminase type 2 (Tg2) and A disintegrin and metalloproteinase with thrombospondin type 1 motif 2 (Adamts 2) that are involved in collagen crosslinking were more induced in old mice [135]. These findings suggest that matrix remodeling during aging is significantly affected and would need to be studied further, in the context of NASH. In addition, the ECM of the aging liver is thought to be less perfused. Mechanical stretching of endothelial cells can induce angiocrine signals that support hepatocyte proliferation and survival [136], and therefore in aging these processes could be impacted leading to decreased regenerative responses.

**Figure 1.**
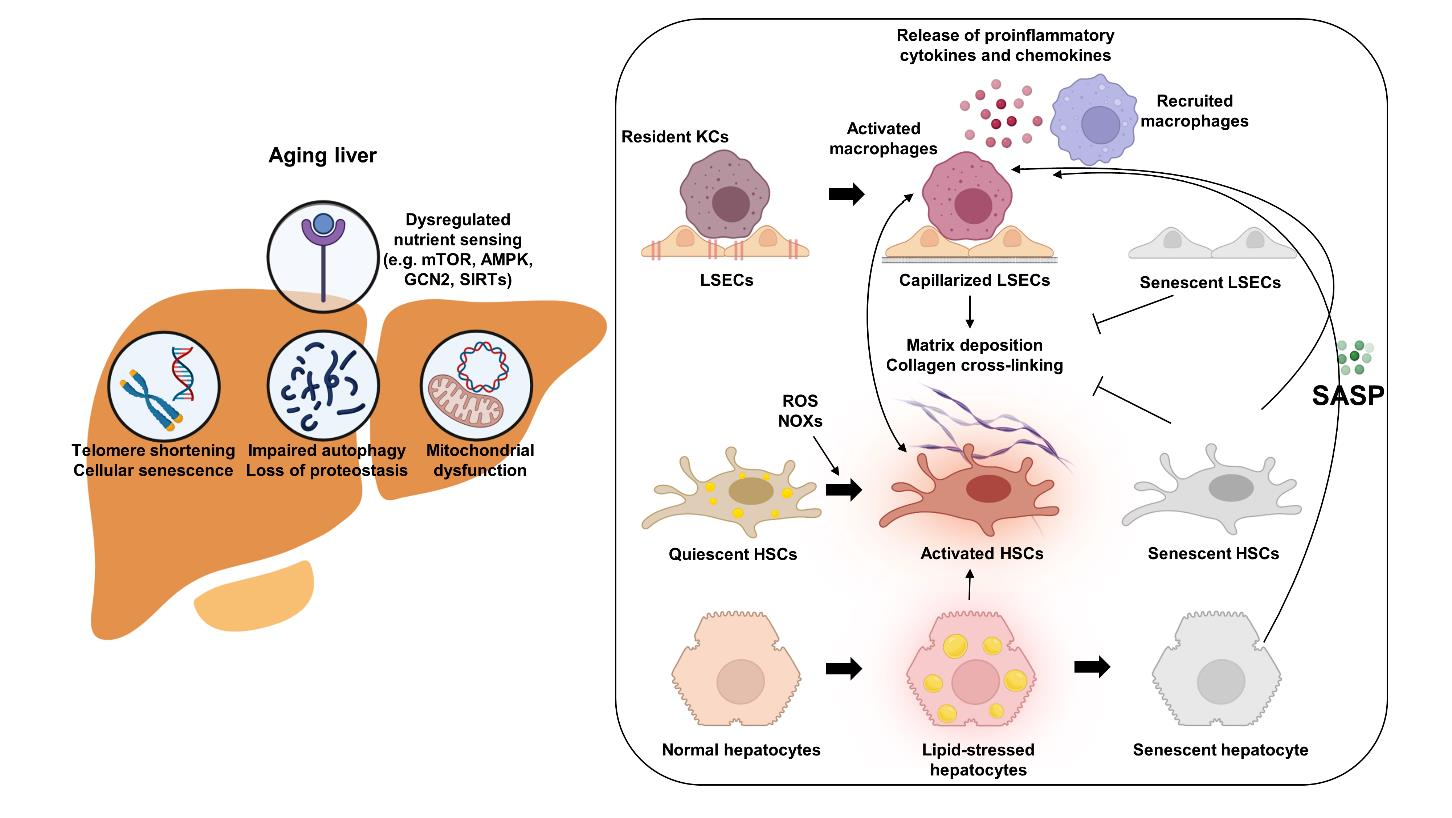
**Schematic summary of aging-related events predisposing to fibrogenic injury in NASH**. Aging-mediated changes in several adaptive pathways exacerbate NASH with enhanced necroinflammation, fibrogenic processes, and reduced fibrolysis. These include cellular senescence, that has distinct, cell type-dependent effects, dysregulated nutrient-sensing pathways (e.g., mTOR, AMPK, GCN2, SIRTs), loss of proteostasis, impaired autophagy, and mitochondrial dysfunction. These either alone or in combination can drive the activation of stellate cells and progression of fibrosis. AMPK, AMP-activated protein kinase; GCN2, general control nonderepressible 2; mTOR, mammalian target of rapamycin; SIRT, Sirtuin; HSC, hepatic stellate cell; KC, Kupffer cell; LSEC, liver sinusoidal endothelial cell; NAFLD, non-alcoholic fatty liver disease; NASH, non-alcoholic steatohepatitis; NOX, NADPH oxidase; ROS, reactive oxygen species; SASP, senescence-associated secretory phenotype.

*LSECs* are important contributors to the fibrogenic process, and development of portal hypertension. LSECs lose their fenestrae and form a basement membrane, called "capillarization", early in the pathogenesis of NASH [137-139]. The LSEC phenotype in aging is linked to the downregulation of vasodilatory pathways (nitric oxide, heme oxygenase), and angiocrine mediators [stabilin-2, CD32b, and vascular endothelial growth factor receptor 2 (VEGFR2)], as well as increased portal pressure [116]. The loss of LSEC fenestrations is also thought to be one of the hallmarks of aging [10], therefore it could be assumed that capillarized LSECs both drive fibrosis and have a decreased ability to clear toxins. For example, oxidized low-density lipoproteins (OxLDLs) and advanced glycation end products-modified proteins are scavenged by LSECs and if they are not properly cleared, this can result in elevated OxLDL levels [140-142] that were found to be increased in the plasma of elderly patients [143]. LSEC senescence is also closely linked to aging. In a recent paper using a tamoxifen-inducible p16 reporter mouse, LSECs were the most positive cells for p16, and the depletion of these cells ameliorated steatosis and inflammation in a NASH model [144]. However, if senescent LSECs are not replaced by non-senescent cells, their removal may activate a fibrogenic response [145]. Various agents were studied and found to increase the fenestrations in LSECs from old mice [10,146-148]. Hunt et al. showed drugs targeting NO, actin, or lipid rafts promote fenestration changes in mouse LSECs [147]. This implies that age-related defenestration can be pharmacologically reversed, which has a potential therapeutic link to dyslipidemia and insulin resistance [147].

## Summary and Future Perspectives

The global increase in aging populations and the epidemics of obesity and T2DM are expected to lead to an exponential increase in NASH in the next decade, and complications of advanced liver disease will disproportionally affect the elderly. Lifestyle changes may cause some improvement in necroinflammatory activity and fibrosis in some patients, but these effects often are not sustained. Novel therapies that address dysregulation of metabolic pathways, increase regenerative capacity in advanced stage disease and/or reduce fibrosis progression are highly needed to improve mortality. While several exciting new modalities are in the pipeline, these are tested in patients of all age groups. Therefore, further studies are needed to evaluate whether these drugs would be effective and well tolerated in the elderly population who often have significant comorbidities. Specific targeting of the primary drivers of age-associated pathways such as senescence and associated SASP activity, deficits in mitochondrial capacity or autophagy/mitophagy, will be as important as directly targeting fibrosis/fibrolysis, and likely only combined strategies will be effective (Fig. 1). Furthermore, improved understanding of the pathobiology of the aging liver through complementary approaches, from patient samples (single cell/nucleus transcriptomics, metabolomics and lipidomics) and models with aged animals will be necessary to define key pathways that could be targeted for reversal of NASH.
